# Identification of non-muscle myosin heavy chain as a substrate for Cdk5 and tool for drug screening

**DOI:** 10.1186/1423-0127-16-55

**Published:** 2009-06-17

**Authors:** Anne Jämsä, Karin Agerman, Ann-Cathrin Radesäter, Jan Ottervald, Jonas Malmström, Gösta Hiller, Gang Liu, Mervi Vasänge

**Affiliations:** 1 AstraZeneca R&D, Forskargatan 20, Building 212, S-151 85 Södertälje, Sweden; 2 Karolinska Institutet, NVS, KI-ADRC, Novum, 5th floor, S-141 57 Huddinge, Sweden

## Abstract

**Background:**

Deregulated activation of cyclin-dependent kinase-5 (Cdk5) is implicated in neurodegenerative disorders such as Alzheimer's disease. One of the restricting factors for developing specific Cdk5 inhibitors is the lack of reproducible and well-characterized cellular in vitro assay systems.

**Methods:**

HEK293 cells were transfected with Cdk5 and its activator p25 as a starting point for an assay to screen for Cdk5 kinase inhibitors. To identify suitable substrates for Cdk5 we utilized an antibody that recognizes phospho serine in a consensus motif for Cdk substrates.

**Results:**

Western blot analysis of transfected cells detected a 200 kDa band that was identified, by mass spectrometry, as non-muscle myosin heavy chain, type B (NMHC-B). Phosphorylation of NMHC-B was evident only in cells that were double transfected with Cdk5/p25 and was dose-dependently inhibited by Roscovitine and other Cdk5 inhibitors. Cdk5 was found to phosphorylate NMHC-B also in the human neuroblastoma SH-SY5Y cell line.

**Conclusion:**

A novel Cdk5 substrate NMHC-B was identified in this study. A cellular assay for screening of Cdk5 inhibitors was established using NMHC-B phosphorylation as a read-out in Cdk5/p25 transfected HEK293 cells. A novel Cdk5 inhibitor was also pharmacologically characterized in this assay system.

## Background

Cyclin-dependent kinase-5 (Cdk5) is a member of the cyclin-dependent kinase (Cdk) family of serine/threonine kinases [1]. Unlike other Cdk's, Cdk5 is not regulated by cyclins and is not involved in cell cycle control. The activity of Cdk5 is regulated by its binding to neuron-specific activator proteins, p35 and p39, [2,3] and by phosphorylation [4]. Although Cdk5 is widely expressed, its kinase activity is detected primarily in the nervous system, mainly because highest expression of its activators is restricted to post-mitotic neurons [5].

Although Cdk5 activity is necessary for many physiological functions and development of the nervous system, deregulated Cdk5 activity is neurotoxic and has been linked to neurodegenerative diseases such as Alzheimer's disease (AD). Conversion of p35 to p25 by the calcium activated protease calpain, is thought to cause deregulation of Cdk5 activity in AD brain [6,7]. The dimeric Cdk5/p25 has been shown to possess prolonged enzymatic activity and potentially alter its cellular localization and substrate specificity of the kinase [6,7]. In AD brain, Cdk5 is thought to hyperphosphorylate tau protein and thus contribute to the formation of neurofibrillary tangles, one of the two major pathological hallmarks of this disease [6-8]. Deregulation of Cdk5 also occurs in other neurodegenerative disorders such as Parkinson's disease [9] and amyotrophic lateral sclerosis [10]. Cdk5 is also implicated in ischemic cell death [11] and contextual fear [12]. Although Cdk5 is crucial for learning and memory, prolonged activity is detrimental and impairs these processes [13-15].

Taken together, data supporting the role of Cdk5 in different pathways connected to pathological processes in the central nervous system is convincing thus making it a potentially important target for drug research. Furthermore, availability of specific and selective Cdk5 inhibitors would enable even more detailed studies on its pathological and biological roles. One of the restricting factors for identifying specific Cdk5 inhibitors is the lack of a reproducible and well-characterized cellular assay system. One of the major reasons is the almost exclusive localization of the active Cdk5/p35(p25) complex to cells of neuronal origin, which makes it difficult to find easy-to-handle cell lines for assay purposes.

We previously investigated retinoic acid and brain-derived neurotrophic factor (RA-BDNF) differentiated SH-SY5Y cells in an attempt to establish a cellular system to study Cdk5 involvement in tau phosphorylation. However, in basal conditions the involvement of Cdk5 in tau phosphorylation is minor [16] and also in stimulated cells increases in tau phosphorylation are very moderate or obscured by the involvement of other kinases [17]. Therefore, we proceeded to investigate HEK293 cells transfected with Cdk5/p25 to identify alternative substrates with a robust phosphorylation signal that would enable characterization of enzyme inhibitors.

We report the establishment of a new cellular screening system, which enables pharmacological characterization of specific Cdk5 inhibitors. In the course of the study, we also identified non-muscle myosin heavy chain, type B (NMHC-B), as a substrate for Cdk5.

## Materials and methods

### Cell cultures, transfections and treatments

#### HEK293 cells

Human embryonic kidney 293 (HEK293) cells were grown in Dulbecco's Modified Eagle Medium (D-MEM, InVitrogen, Sweden) with 4.5 g/l glucose, 2 mM glutamine and 110 mg/l sodium pyruvate. The medium was supplemented with 1% non-essential amino acids (InVitrogen, Sweden) and 10% heat-inactivated Fetal Calf Serum (FCS, HyClone, Logan, Utah, USA). For transfection experiments, the cells were plated at a density of 2.0 × 10^5 ^cells/cm^2 ^in 6-well culture dishes (Corning, Lowell, MA, USA). Day 1 after plating, the cells were transfected with equal amount of p25 plasmid (pAPC227, Molecular Pharmacology, AstraZeneca R&D, Södertälje, Sweden) and Cdk5 plasmid (pAPC226, Molecular Pharmacology, AstraZeneca R&D, Södertälje, Sweden), 1.5 μg each. Lipofectamine™2000 (InVitrogen, Sweden) was used as a transfection reagent. Lipofectamine™2000 (7.5 μl/transfection) was first diluted in cell culture medium without FCS and incubated for 5 min at RT. The plasmid DNA diluted in medium was then combined with Lipofectamine and incubated for further 20 min at RT. The complexes were added to the cells and the transfection was carried out for 24 hours. Treatment with Cdk5 inhibitors was carried out during the last 4 hours of transfection.

The p25 and Cdk5 genes were cloned into mammalian expression vectors, pcDNA3 and pcDNA3.1(-) (Molecular Pharmacology, AstraZeneca R&D, Södertälje, Sweden), respectively and the expression was under the control of CMV promoter. Cdk5 inhibitors used in this study were Roscovitine (Sigma, Sweden), 7-ethyl-4-[(4-fluorophenyl) amino]-3,5,7-triaza bicyclo [4.4.0.] deca-1,3,5,9-tetraen-8-one from Warner Lambert company (WL compound)(Medicinal Chemistry, AstraZeneca R&D, Södertälje, Sweden) [18,19] and 4-(6-chlorobenzothiazol-2-yl)thiophene-2-sulfonamide (AZ compound)(Medicinal Chemistry, AstraZeneca R&D, Södertälje, Sweden) [20]. Stock solutions of Cdk5 inhibitors were prepared in dimethyl-sulfoxide (DMSO). All cultures including the control cells received equal amounts of DMSO, the final concentration being 0.158%.

#### SH-SY5Y cells

SH-SY5Y cells were grown in medium with equal amount of Minimum Essential Medium (MEM, InVitrogen, Sweden) and Nutrient Mixture Ham's F-12 (InVitrogen, Sweden), supplemented with 1% non-essential amino acids and with heat-inactivated FCS. Cells were plated at a density of 4.0 × 10^4 ^cells/cm^2 ^in 6-well culture dishes using cell medium with 10% FCS. For differentiation, the cells were treated 6 days with 10 μM all-trans-retinoic acid (RA, Sigma, Sweden) in the medium containing 1% FCS. In some experiments the cells were left undifferentiated and these cells were cultured in medium containing 10% FCS. SH-SY5Y cells have high endogenous levels of Cdk5 and therefore only the p25 plasmid (pAPC227) was transfected to the cells. Transfections were carried out in the same manner as for HEK293 cells except that 10 μl of Lipofectamine™2000 was used for each transfection with 8 μg p25 plasmid.

### Western blot

HEK293 cells were lysed in buffer containing 10 mM Tris-HCl pH 7.2, 150 mM NaCl, 2 mM EDTA, 50 mM NaF, 1 mM Na_3_O_4_V, 0.5% NP-40 and 1 complete protease inhibitor cocktail tablet (Roche Diagnostics Scandinavia AB, Sweden)/10 ml buffer. Lysis buffer for SH-SY5Y cells contained 50 mM Tris-HCl pH 8.0, 150 mM NaCl, 1 mM EDTA, 10 mM NaF, 1 mM Na_3_VO_4_,1% Triton X-100 and 1 complete protease inhibitor cocktail tablet/10 ml buffer. Cell lysates were centrifuged at 14 000 rpm (Eppendorf 5417R, Germany) for 15 min. The protein content in the supernatants was measured using BCA Protein Assay kit (Pierce Biotechnology, Rockford, IL, USA). Samples containing 40–50 μg protein were resolved in 10% NuPage^®^Bis-Tris gels (Invitrogen, Sweden) and the proteins were transfered to Hybond nitrocellulose membranes (Amersham Biosciences, Sweden). Membranes were blocked in PBS with 0.05% Tween 20 containing 5% nonfat dry milk for 1 hour at room temperature (RT). Primary antibodies were diluted in either 5% BSA or 5% milk and incubations were carried out at 4°C over night. Primary antibodies were used at the following dilutions: Cdk5 (C-8; Santa Cruz Biotechnology Inc., Santa Cruz, California, USA) 1:2000, p35 (C-19; Santa Cruz Biotechnology Inc., Santa Cruz, California, USA) 1:2000, pSer Cdk substrate (Cell Signaling Technology Inc., Danvers, Massachuesetts, USA) 1:800 and non-muscle heavy chain myosin [3H2] (abcam, UK) 1:1000. Horseradish-peroxidase (HRP) conjugated secondary antibodies (Amersham Biosciences, Sweden) were incubated 1 hour at RT in 5% milk at the dilution of 1:5000 for anti-rabbit and 1:10 000 for anti-mouse antibody. Blots were developed using the enhanced chemiluminescence (ECL) Western blotting detection system (Amersham Biosciences, Sweden). When needed, membranes were stripped with Restore Western blot stripping buffer (Pierce Biotechnology, Rockford, IL, USA) for 30 min at 50°C. Average density of the bands was measured in Fluor-S™MultiImager (Bio-Rad Laboratories AB, Sweden) by using Quantity One software. The inhibition curves were analysed by non-linear regression using Graph Pad Prism.

### Immunoprecipitation

200 μg of protein in 100 μl lysis buffer was precleared for 1 hour at 4°C with 10 μl protein A/G Plus agarose beads (Santa Cruz Biotechnology Inc., Santa Cruz, California, USA). The samples were centrifuged at 14 000 rpm (Eppendorf 5417R) for 1 min and the supernatants were transferred to new tubes. 10 μl of pSer Cdk substrate antibody was added to the supernatants and incubated rotating over night at 4°C, 20 μl protein A/G Plus agarose beads was added and incubated further for 1 hour. The samples were centrifuged at 6000 rpm (Eppendorf 5417R) for 2 min. The supernatants were discarded and the pellets washed three times in 150 μl lysis buffer. The immunoprecipitates were then diluted in lysis buffer and processed for Western blot analysis.

### Mass spectrometry and sequence analysis

Protein spots of interest were excised from gels using a spot cutter robot (Bio-Rad, Hercules, CA, USA) and transferred to 96-well plates. The gel plugs were transferred to eppendorf tubes approximately 10 pieces in each tube. Sypro Ruby^® ^(Molecular Probes, the Netherlands)-stained gel pieces were manually treaded as follows; wash with 100 μl ddH_2_O three times for 15 min, followed by wash with 25 mM ammoniumbicarbonate/acetonitril (1:1 v/v) three times for 15 min. Thereafter acetonitril was added to cover the gel pieces to shrink them down. The gel pieces were rehydrated by incubating with 50 μl 25 mM ammoniumbicarbonate for 5 min, adding acetonitril and incubating for another 15 min. Thereafter all liquid was removed and the gel pieces were dried completely in vacuo. The samples were placed on ice and a freshly prepared and chilled digestion solution containing 10 ng/μl of trypsin (Promega, Madison, WI, USA) in 25 mM ammoniumbicarbonate was added to cover the gel pieces. Incubation on ice for 10 min and in RT for 30 min was carried out and more buffer was added if necessary. The samples were then placed on a heater at 37°C over night. One-hour incubation with equal amount of 1% formic acid stopped the reaction. To concentrate and desalt the samples ZipTip (Millipore, Billerica, Massachuesetts, USA) was used according to manufacturers descriptions. Samples were then washed twice with 1% formic acid and eluted with 2 μl of matrix solution α-cyano-4-hydroxycinmic acid (Waters Corporation, Milford, Massachusetts, USA) direct onto 96-position MALDI target plate (Waters Corporation, Milford, Massachusetts, USA) and crystals of matrix and peptide were formed. After MALDI-ToF (Waters Corporation, Milford, Massachusetts, USA) was used to attain the mass of each peptide and the resulting peaklist was imported into the search engineers Protein Lynx Global Server 2 (PLGS2, Waters Corporation, Milford, Massachusetts, USA) and MASCOT (Matrix Sciences Ltd, UK). Several databases were selected as information sources.

### Scintillation Proximity Assay

The assay experiments were carried out in duplicate with 10 different concentrations of the inhibitors in clear-bottom 384-well microtiter plates (Item No 3706, Corning, Lowell, Massachusetts, USA). Recombinant human Cdk5/p25 (Biotech Laboratory, AstraZeneca R&D, Södertälje, Sweden) was added at a final concentration of 3.3 nM in an assay buffer containing 23.3 mM HEPES, pH 7.35, 0.2 mM EDTA, 12.5 mM KCl, 10 mM β-glycerophosphate, 0.02% β-mercaptoethanol, 0.007% Brij 35, 0.8% glycerol and 0.05% BSA. After incubation for 15 min the reaction was initiated by the addition of 2.95 μM (final concentration) of a biotinylated peptide substrate, Biotin-Ala-Lys-Lys-Pro-Lys-Thr-Pro-Lys-Lys-Ala-Lys-Lys-Leu-OH (Bachem, Switzerland), 0.07 μCi [γ^33^P]ATP (Amersham, UK), 2 μM unlabelled ATP and 10 mM MgCl_2 _in an final assay volume of 21 μl. After incubation for 40 min at RT, each reaction was terminated by the addition of 30 μl stop solution containing 24 mM EDTA, 2.2 mM ATP and 0.225 mg streptavidine coated SPA beads (Amersham, UK). The microtiter plates were centrifuged for 2 minutes at 200 g and the radioactivity was determined in a liquid scintillation counter (1450 MicroBeta Trilux, Wallac, Finland). The inhibition curves were analysed by non-linear regression using Graph Pad Prism.

## Results

### Transfection of HEK293 cells with Cdk5/p25 reveals one phosphoprotein that can be inhibited with Roscovitine

To identify novel Cdk5 substrates suitable for characterising enzyme inhibitors, we transfected HEK293 cells with Cdk5 and p25 to get an active Cdk5/p25 complex. Cell lysates from transfected cells were analyzed with Western blotting using an antibody that recognizes phospho serine in a consensus motif for Cdk substrates.

**Figure 1:**
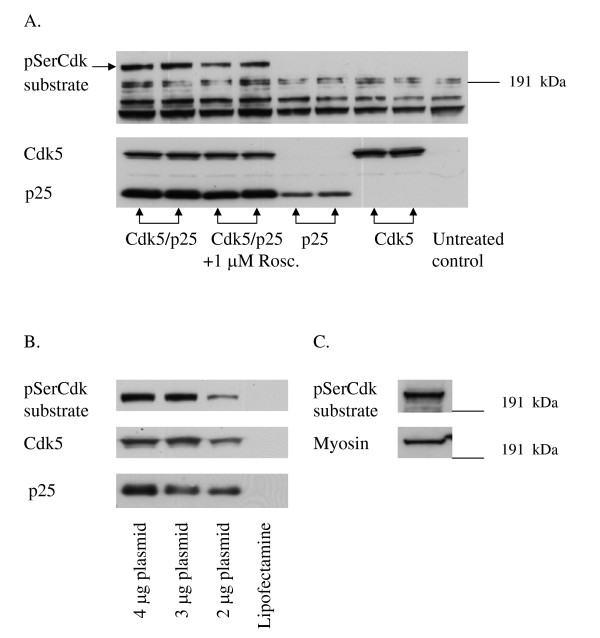
**Transfection of Cdk5/p25 into HEK293 cells identifies NMHC-B as a substrate for Cdk5**. **A**. Western blot showing pSerCdk substrate, Cdk5 and p25 levels in cells transfected with Cdk5/p25, Cdk5/p25 transfected cells treated with 1 μM Roscovitine, cells transfected only with p25 or Cdk5 and untransfected cells. A 200 kDa band appeared only in the Cdk5/p25 double transfected cells. This band decreased in intensity when treated with 1 μM Roscovitine and did not show up in single transfected or untransfected cells. **B**. Western blot showing pSerCdk substrate, Cdk5 and p25 levels in cells transfected with 2, 1.5 or 1 μg of each plasmid and in cells treated only with transfection reagent, Lipofectamine. The cells transfected with 1.5 μg of Cdk5 and p25 plasmid, i.e.3 μg total plasmid DNA, showed sufficient phosphorylation of Cdk substrates and least effect on cell morphology and viability (data not shown). **C**. Western blot showing a pSerCdk antibody immunoprecipitated sample from Cdk5/p25 transfected HEK293 cells probed with pSerCdk substrate antibody and with antibody to NMHC-B. Detection of the same band by both antibodies verifies that the 200 kDa band is NMHC-B.

The pSerCdk substrate antibody recognized several phosphoproteins. A band with molecular weight of approximately 200 kDa appeared in cells that were double transfected with Cdk5 and p25 but did not show up in non-transfected or single transfected (only Cdk5 or p25) cells (Fig. 1A). The 200 kDa band decreased in intensity in Cdk5/p25 transfected cells treated with 1 μM Roscovitine (Fig. 1A). The other bands were visible even with single transfection and were not inhibited by Roscovitine. We therefore concluded that only the 200 kDa band is regulated by Cdk5.

In initial studies, the HEK293 cells were transfected with 1, 1.5 or 2 μg of Cdk5 and p25 plasmids. The setting where the cells were transfected with 1.5 μg of each plasmid, i.e. 3 μg total plasmid DNA, showed least effect on cell morphology and viability (data not shown) with sufficient phosphorylation of Cdk substrates (Fig 1B). This plasmid concentration was therefore chosen for all further experiments.

### Cdk5 phosphorylates non-muscle myosin heavy chain in Cdk5/p25 transfected HEK293 cells

Mass spectrometry identified the 200 kDa band as a non-muscle myosin heavy chain, type B (NMHC-B) (GenBank access number P35580). The total number of mass values matched was 130 and the sequence coverage 57%. Verification that the 200 kDa pSerCdk substrate band indeed was myosin was done by immunoprecipitation. When Cdk5/p25 transfected samples were immunoprecipitated with pSerCdk substrate antibody and then run on Western blot, the pSerCdk substrate antibody and NMHC-B antibody detected the same band (Fig 1C). Western blot analysis of cell lysates gave the same results (data not shown).

### Cdk5 inhibitors dose-dependently inhibit peptide substrate phosphorylation in a Scintillation proximity assay

**Figure 2:**
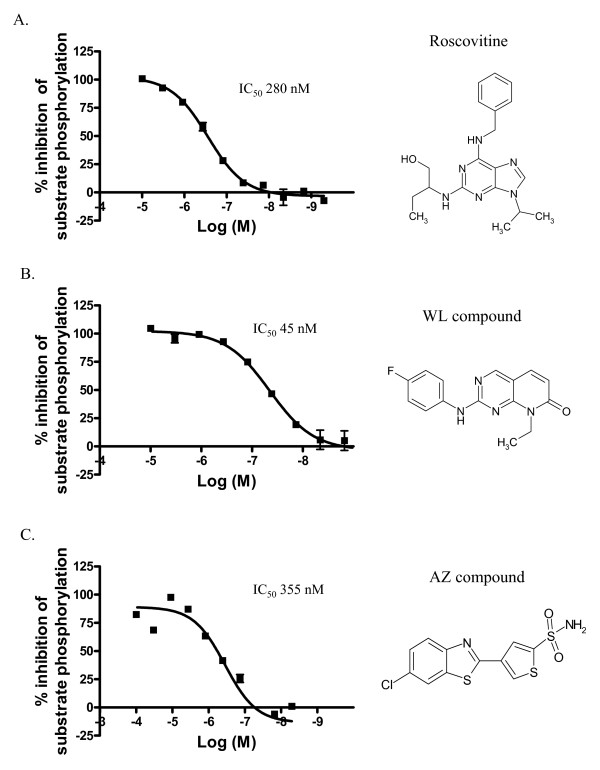
**Cdk5 inhibitors dose-dependently inhibit peptide substrate phosphorylation in a SPA assay**. **A**. Roscovitine dose-dependently inhibits substrate phosphorylation with IC_50 _280 nM. **B**. WL compound dose-dependently inhibits substrate phosphorylation with IC_50 _45 nM. **C**. AZ compound dose-dependently inhibits substrate phosphorylation with IC_50 _355 nM. Structures of these compounds are shown next to their respective inhibition curves.

Initial identification of potent Cdk5 inhibitors was done in a Scintillation Proximity Assay (SPA). This biochemical assay measures inhibition of the recombinant human Cdk5/p25 protein kinase by different compounds with a synthetic peptide as a substrate. Several compounds displayed a potency in the nanomolar range and were further tested in Cdk5/p25 transfected HEK293 cells. Figure 2 shows structures and SPA results from three representative compounds, Roscovitine, a WL compound and 4-(6-chlorobenzothiazol-2-yl)thiophene-2-sulfonamide (an AZ compound). IC_50 _values in SPA assay were 280 nM for Roscovitine (Fig 2A), 45 nM for the WL compound (Fig 2B) and 355 nM for the AZ compound (Fig 2C).

### Cdk5 inhibitors dose-dependently inhibit non-muscle myosin heavy chain phosphorylation in HEK293 cells

To further investigate Cdk5 involvement in NMHC phosphorylation, we used the three pharmacological inhibitors of Cdk5 and analyzed NMHC phosphorylation by Western blot using the pSerCdk substrate antibody.

**Figure 3:**
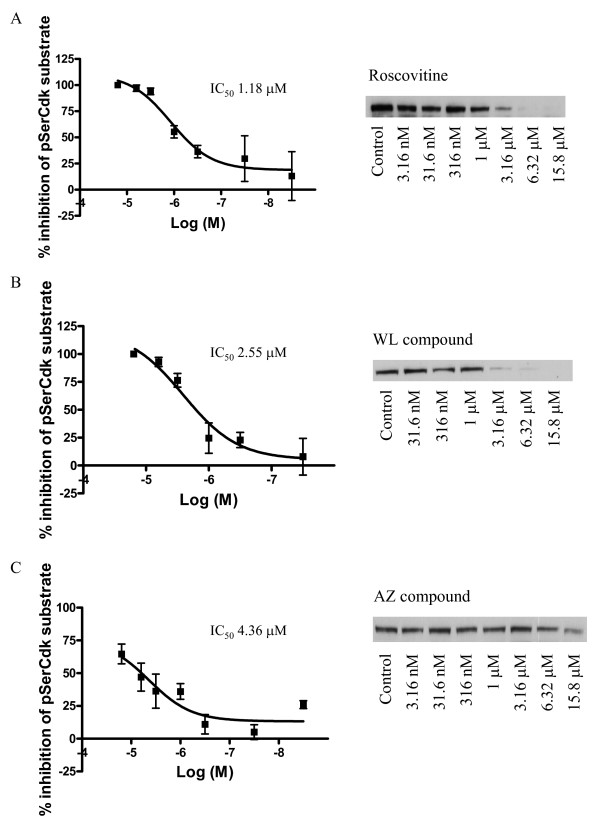
**Cdk5 inhibitors dose-dependently inhibit NMHC-B phosphorylation in Cdk5/p25 transfected HEK293 cells**. **A**. Roscovitine dose-dependently inhibits pSerCdk phosphorylation with IC_50 _1.18 μM. **B**. WL compound dose-dependently inhibits pSerCdk phosphorylation with IC_50_2.55 μM. **C**. AZ compound dose-dependently inhibits pSerCdk phosphorylation with IC_50 _4.36 μM. A Western blot of pSerCdk substrate in control and in cells treated with different concentrations of inhibitor is shown next to their respective inhibition curves.

The Cdk5 inhibitors Roscovitine, WL and AZ compounds concentration-dependently inhibited pSerCdk substrate phosphorylation with IC_50 _of 1.18 μM, 2.55 μM and 4.36 μM, respectively. (Fig 3A, B, C). As a comparison, a non-kinase inhibitor 5-(4-chlorobenzoyl)-2-hydroxy-6-methyl-3-tert-butyl-benzoic acid (Medicinal Chemistry, AstraZeneca R&D, Södertälje, Sweden) did not show inhibition of pSerCdk substrate phosphorylation (data not shown).

### Cdk5 phosphorylates non-muscle myosin heavy chain in human neuroblastoma SH-SY5Y cells

**Figure 4:**
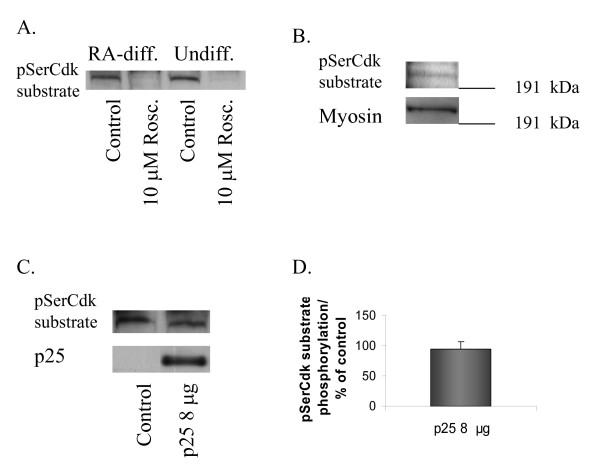
**Cdk5 phosphorylates NMHC-B in SH-SY5Y cells**. **A**. A Western blot showing a 200 kDa pSerCdk substrate band in undifferentiated and RA-differentiated SH-SY5Y cells. Phosphorylation of pSerCdk substrate is completely inhibited when cells are treated with 10 μM Roscovitine for 4 hours. **B**. A Western blot showing a pSerCdk antibody immunoprecipitated sample from SH-SY5Y cells probed with pSerCdk substrate antibody and with antibody to NMHC-B. Detection of the same band by both antibodies verifies that a 200 kDa band found in SH-SY5Y cells is NMHC-B. **C**. A Western blot showing pSerCdk substrate and p25 levels in control and in p25 transfected SH-SY5Y cells. Transfection with p25 does not increase pSerCdk substrate phosphorylation in SH-SY5Y cells. **D**. Densitometric quantification of pSerCdk phosphorylation in SH-SY5Y cells transfected with 8 μg p25 plasmid presented as % of control (mean ± SEM).

We also investigated NMHC-B phosphorylation by Cdk5 in human neuroblastoma SH-SY5Y cells. The 200 kDa pSerCdk substrate band was detected both in undifferentiated and RA-differentiated SH-SY5Y cells and phosphorylation could be completely inhibited with 10 μM Roscovitine (Fig. 4A). Verification that the 200 kDa pSerCdk band detected in SH-SY5Y cells was in fact NMHC-B was done by immunoprecipitation. When samples from undifferentiated SH-SY5Y cells were immunoprecipitated with pSerCdk antibody and then run on Western blot, pSerCdk antibody and NMHC-B antibody detected the same band (Fig. 4B). pSerCdk substrate phosphorylation did not increase in intensity in p25 transfected cells, either in RA-differentiated (Fig. 4C, D) or undifferentiated (data not shown).

## Discussion

As Cdk5 kinase activity is almost exclusively restricted to neuronal post-mitotic cells, it has been challenging to identify both a specific and robust cellular assay to enable pharmacological characterization of enzyme inhibitors. In an attempt to identify a suitable system, we set up to transfect HEK293 cells, with both the Cdk5 enzyme and its activator p25.

Previously, a wide variety of substrate proteins have been identified for Cdk5, which reflects its role in diverse functions. For instance, Cdk5 is involved in regulation of cytoskeletal dynamics by phosphorylating neurofilaments and microtubule-associated proteins tau and MAP1B [21-23]. Cdk5 participates in synaptic function and neurotransmission by phosphorylating many synaptic proteins [24]. In addition, Cdk5 has an important function in the development of the central nervous system regulating neuronal migration [25] and axon guidance [26].

As Cdk5 phosphorylates serines and threonines immediately upstream of a proline residue [27], an antibody that recognizes phospho serine in this consensus motif for Cdk substrate, was utilized in our study. As a result, a non-muscle myosin heavy chain, type B (NMHC-B) was identified as a Cdk5 substrate. Myosins constitute a large family of actin-based motor proteins [28] and non-muscle myosins are composed of two heavy chains and two pairs of light chains. In contrast to muscle myosins, which form stable myofibrils, the non-muscle myosin constitutes part of the actinomyosin cytoskeleton. Both actin and myosin in non-muscle cells can be rapidly assembled or disassembled in response to extracellular signals to allow cell shape changes needed for movement, cell division or secretion [29,30]. Functional activities of myosin are regulated by light chain and heavy chain phosphorylation [29].

In vertebrates, NMHC are phosphorylated by kinases such as Protein Kinase C, Casein kinase II and Ca^2+^/calmodulin-dependent protein kinase [31,32]. Phosphorylation of NMHC by Cdk5 has not been demonstrated previously although a link between Cdk5 and non-muscle myosin was recently discovered. Ledee et al. [33] showed a specific interaction between Cdk5 activator p39 and non-muscle myosin essential light chain.

To test the physiological relevance of our finding, NMHC-B phosphorylation by Cdk5 was investigated in a second cell line, a human neuroblastoma SH-SY5Y. HEK293 cells only express Cdk5 at low levels and they lack endogenous Cdk5 activity, whereas SH-SY5Y cells express both Cdk5 and its activator p35 and exhibit basal Cdk5 activity [17]. NMHC-B phosphorylation by Cdk5 was also detected in SH-SY5Y cells, both in undifferentiated and RA-differentiated ones. However, when p25 was transfected to the cells, no increase in phosphorylation of NMHC-B was observed in contrast to transfected HEK293 cells where phosphorylation was dose-dependent on plasmid concentration. This could be due to the fact that NMHC-B is already maximally phosphorylated by endogenous Cdk5 kinase activity.

In drug discovery research, it is customary that the first filtering assay used in compound characterization is a fast, readily reproducible, miniaturizable and in most cases biochemical assay. The primary purpose for this system is to serve as a means of ranking the compounds according to potency. Thereafter, the chemically most interesting and potent compounds need to be further characterized in more complex cellular environment. For Cdk5/p25 we describe here a HEK293 cell system with quantification of NMHC-B phosphorylation as read-out. The compounds tested in the present setting represent different chemical classes, with the known Cdk inhibitor, Roscovitine, a Warner-Lambert reference compound, and a compound with an additional distinct structural class identified in AstraZeneca. We also included a Cdk5 inactive compound in order to demonstrate specificity of the measured signal. All the three compounds demonstrated clear concentration dependent response, the IC_50 _values, as expected, being higher than in the SPA based biochemical assay. The magnitude of drop-offs between the assays for the different chemical classes varied, which is a relatively common phenomenon and can most likely be explained at least partly by differences in their physical and chemical properties, as well as in cell permeability. The concentration response curve for the AZ compound is not well defined in the higher concentration area, which can also be seen in the biochemical assay. This is likely to be due to the poor solubility of the compound leading to precipitation at higher concentrations.

The AZ compound used in this study is a novel type of kinase inhibitor (Malmström and Viklund 2006). A close analogue to it was co-crystallised with the Cdk5/p25 complex and the X-ray structure showed that the ligand is not directly bound to the backbone (Glu 81 and Cys83) in the ATP site of the kinase as is the usual case. Instead, a water molecule was found to form a bridging interaction between the ligand and the hinge backbone (Malmström et al., unpublished results). The AZ compound is assumed to bind in a similar fashion, although X-ray crystallography would be required to confirm this.

Phosphorylation of NMHC-B was evident only in Cdk5/p25 double transfected cells indicating that only Cdk5 phosphorylates this substrate in HEK293 cells. Cdk5 inhibitors concentration-dependently inhibited NMHC-B phosphorylation and at higher concentrations phosphorylation was totally blocked. Described assay with NMHC-B phosphorylation as a read-out is thus very specific and sensitive. However, a limitation of the system at present is its relatively low throughput caused by the Western blot based quantification of the phosphorylation signal. An attempt to solve this could for instance be use of Image analysis in a high content screening setting such as ImageXpress.

## Conclusion

A novel Cdk5 substrate, NMHC-B, was identified in this study. Using phosphorylation of this substrate as a read-out, we developed a specific and sensitive cellular assay that can be used for validation of compounds designed to inhibit Cdk5. A novel Cdk5 inhibitor was also pharmacologically characterized in this assay system.

## Abbreviations

AD: Alzheimer's disease; Cdk5: cyclin-dependent kinase; HEK293: human embryonic kidney 293; NMHC-B: non-muscle myosin heavy chain, type B; RA: retinoic acid; SPA: Scintillation proximity assay.

## Competing interests

The authors declare that they have no competing interests.

## Authors' contributions

AJ carried out experiments in SH-SY5Y cells, participated in design of the study and drafted the manuscript. KA, ACH and AJ all participated in experiments with HEK293 cells. KA also participated in the design of the study and edited the manuscript. JO carried out the sequence analysis using mass spectrometry and protein identification using Mascot server. GH carried out SPA assays. JM was responsible for the design and synthesis of the novel Cdk5 kinase inhibitor used in the study. GL designed the plasmids used in transfection experiments and participated in the initial transfection experiments in HEK293 cells. MV organized the study, participated in study design and coordination and revised the manuscript. All authors read and approved the final manuscript.
